# Global analysis of fungal morphology exposes mechanisms of host cell escape

**DOI:** 10.1038/ncomms7741

**Published:** 2015-03-31

**Authors:** Teresa R. O’Meara, Amanda O. Veri, Troy Ketela, Bo Jiang, Terry Roemer, Leah E. Cowen

**Affiliations:** 1 Department of Molecular Genetics, University of Toronto, Toronto, Ontario, Canada M5S 1A8; 2 Bioprocess Technology & Expression, Merck Research Laboratories, 2000 Galloping Hill Rd, Kenilworth, New Jersey 07033, USA; 3 Department of Infectious Diseases, Merck Research Laboratories, 2000 Galloping Hill Rd, Kenilworth, New Jersey 07033, USA

## Abstract

Developmental transitions between single-cell yeast and multicellular filaments underpin virulence of diverse fungal pathogens. For the leading human fungal pathogen *Candida albicans,* filamentation is thought to be required for immune cell escape via induction of an inflammatory programmed cell death. Here we perform a genome-scale analysis of *C. albicans* morphogenesis and identify 102 negative morphogenetic regulators and 872 positive regulators, highlighting key roles for ergosterol biosynthesis and N-linked glycosylation. We demonstrate that *C. albicans* filamentation is not required for escape from host immune cells; instead, macrophage pyroptosis is driven by fungal cell-wall remodelling and exposure of glycosylated proteins in response to the macrophage phagosome. The capacity of killed, previously phagocytized cells to drive macrophage lysis is also observed with the distantly related fungal pathogen *Cryptococcus neoformans*. This study provides a global view of morphogenetic circuitry governing a key virulence trait, and illuminates a new mechanism by which fungi trigger host cell death.

## Abstract (web summary)

Several pathogenic fungi such as *Candida albicans* undergo transitions between single-celled forms and multicellular filaments. Here the authors perform a genome-scale analysis of *C. albicans* and show that, contrary to common belief, filamentation is not required for escape from host immune cells.

## Graphical abstract

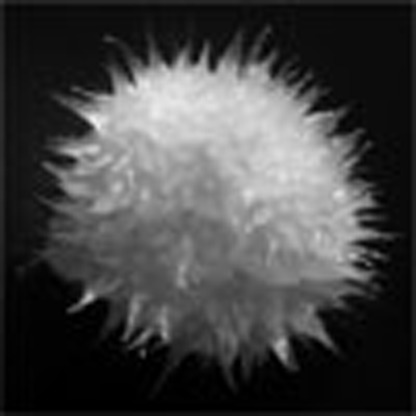

The capacity to transition between distinct morphological states in response to environmental cues is a broadly conserved trait in the fungal kingdom, with profound impacts on diverse facets of biology^1^. For *Candida albicans*, a leading fungal pathogen of humans, the ability to transition between yeast and filamentous forms is a key virulence trait. Most mutants that are locked in either growth state are avirulent in mouse models of systemic candidiasis^2^,^3^,^4^. The current paradigm is that yeast cells are critical for colonization, early infection and dissemination, while filaments are responsible for tissue invasion and deep-seated infection^2^,^4^,^5^,^6^. Filaments are further implicated in virulence as they express virulence factors such as adhesins and proteases, and are thought to be required for escape from host immune cells^6^,^7^.

The reversible morphological transition from yeast to filaments is induced by diverse cues including serum, nutrient limitation and elevated carbon dioxide levels^7^,^8^. The regulatory circuitry governing morphogenesis in response to these cues is complex, and the molecular mechanisms underpinning much of the morphogenetic programme remain enigmatic^9^. A recent genome-scale analysis of morphogenesis in *Saccharomyces cerevisiae* identified many novel regulators of haploid invasive growth, biofilm formation and diploid pseudohyphal growth, providing insight into the regulatory circuitry governing filamentation in the model yeast^10^. The study also assessed morphology of 829 *C. albicans* mutants, and established that despite some conservation in morphogenetic regulators between the two species, there was substantial rewiring over evolutionary time^10^. Currently, 73% of the 6,354 predicted genes in the *C. albicans* genome remain uncharacterized, and ~40% of these genes lack identifiable homologues in *S. cerevisiae*^6^,^11^. Further, model yeasts cannot provide a surrogate for analysis of the impact of morphogenesis on interactions with host immune cells^8^,^12^. The complexities of regulatory circuitry governing *C. albicans* morphogenesis necessitate a functional genomic analysis of filamentous growth directly in the pathogen.

Here we leverage a genome-scale *C. albicans* mutant library to identify regulators of morphogenesis in response to host-relevant environmental cues. In the HET (heterozygous deletion) collection, a uniquely double-barcoded selectable marker cassette replaces one copy of every targeted gene in 5,296 heterozygous diploid strains^5^,^9^. The GRACE (gene replacement and conditional expression) collection consists of 2,356 HET strains where the expression of the remaining wild-type allele of a gene is governed by a tetracycline-repressible promoter^7^,^10^. As a primary approach, we perform imaging-based arrayed screens to systematically assess the capacity of each GRACE strain to filament in response to serum upon transcriptional repression of the target gene by the tetracycline analogue, doxycycline (DOX). In conjunction, we develop and validate a novel and complementary pooled screening approach to facilitate rapid identification of filamentation-deficient *C. albicans* mutants. Through these methods, we identify 872 genes that are required for morphogenesis in *C. albicans* in response to serum, and 102 genes that encode repressors of filamentation. Analysis of the interaction between *C. albicans* mutants blocked in filamentation and mammalian macrophages reveals that filamentation is not required for escape from macrophages. Rather, *C. albicans* responds dynamically to the macrophage phagosome to expose cell-wall glycosylated proteins that are necessary to induce macrophage lysis. Moreover, we find that *Cryptococcus neoformans* also remodels its cell surface to induce host cell lysis, suggesting a broadly conserved mechanism among fungal pathogens.

## Results

### Defining the *C. albicans* essential gene set

**Figure 1:**
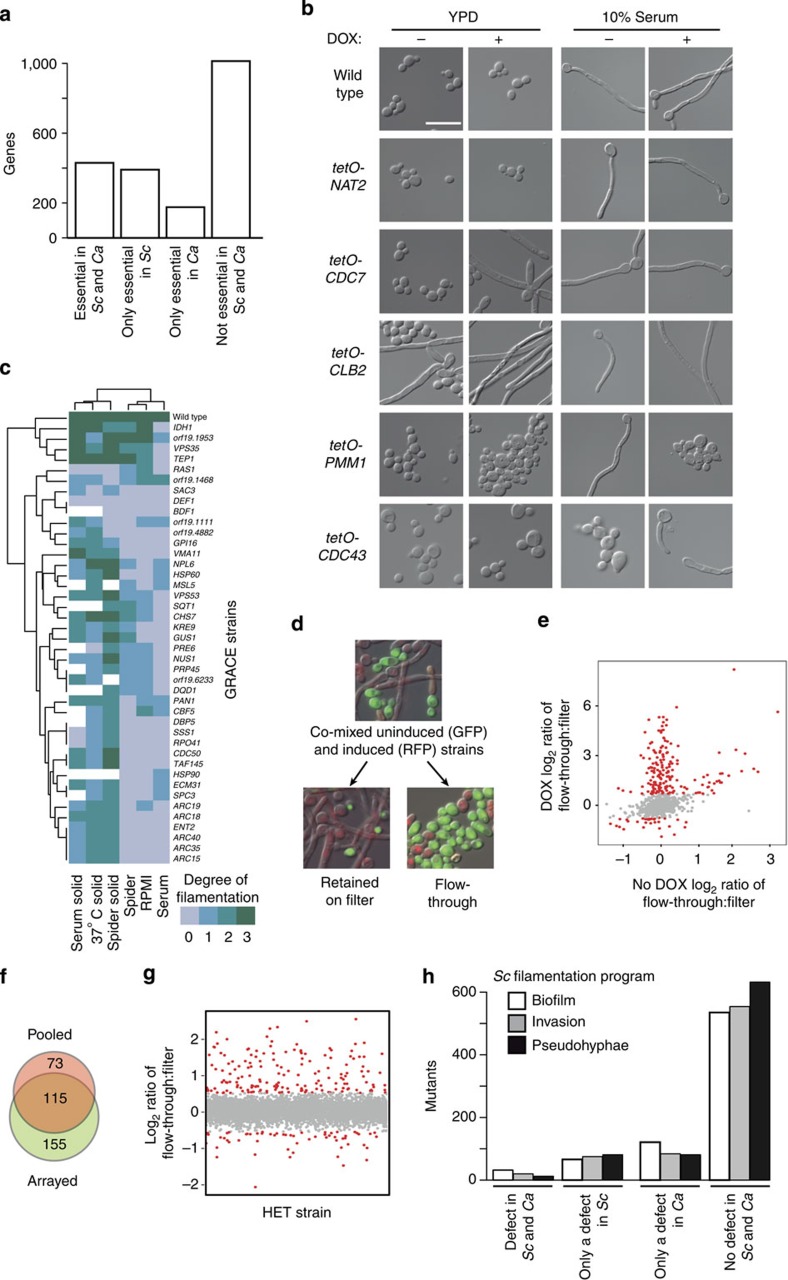
Functional genomics analysis of *C. albicans* morphogenesis. (**a**) Comparison of essentiality between *S. cerevisiae* (*Sc*) and *C. albicans* (*Ca*) genes. Each count represents a gene with direct homology between the two species. (**b**) Representative images of phenotypes identified in the arrayed screen. Scale bar, 20 μm. (**c**) Clustering analysis of GRACE strains and phenotypes observed in distinct filament-inducing conditions with 0.05 μg ml^−1^ DOX. White boxes indicate a lack of growth in that condition. Clustering was performed using the Heatmap function in R. (**d**) Yeast expressing green fluorescent protein (GFP) and filaments expressing red fluorescent protein (RFP) can be separated by filtration using a 10 μm filter. (**e**) Scatterplot of normalized barcode read log_2_ fold difference between flow-through and filter-retained GRACE strains in pooled filtration assays. Red dots represent GRACE strains with ≥4 MADs in log_2_ fold change flow-through:filter retained in the DOX population (significant change in filamentation). (**f**) Venn diagram depicting overlap in barcoded GRACE hits in the arrayed and pooled morphology screens (non-barcoded hits in arrayed screen omitted). (**g**) Scatterplot of barcode reads for the serum-exposed HET filtration screen. Red dots represent ≥4 MAD log_2_ fold difference between flow-through and filter-retained normalized reads. (**h**) Comparison of filamentation programmes between *S. cerevisiae* (*Sc*) and *C. albicans* (*Ca*). Each count represents a gene with direct homology between the two species. Filamentation data for *S. cerevisiae* are from the study by Ryan *et al.*^10^.

The GRACE collection is enriched for homologues of genes that are known to be essential in *S. cerevisiae* and other fungi^7^,^10^. We screened the GRACE collection of 2,356 strains to define the essential gene set within it and identified a total of 634 strains that exhibited severe growth defects or death following target gene transcriptional repression with 100 μg ml^−1^ DOX (Supplementary Data 1). When comparing essential genes between *S. cerevisiae* and *C. albicans* (Fig. 1a), we found that although there was a strong correlation between the species (*P*<0.001, Fisher’s exact test), the positive predictive value from the *S. cerevisiae* essentiality was only 52.4%, emphasizing the importance of examining gene function in the organism of interest. Since the GRACE set is already enriched for homologues of known fungal essential genes, the predictive power of *S. cerevisiae* across the remaining *C. albicans* gene set is likely to be much lower. To uncover morphology defects in GRACE strains, we used 0.05–5 μg ml^−1^ DOX, which is generally sufficient to repress target gene transcription and enable analysis of essential gene function with minimal off-target effects (Supplementary Figs 1 and 2).

### Global analysis of morphogenesis

We surveyed the morphology of each GRACE strain in response to four combinatorial conditions of incubation with and without 10% serum, and with and without DOX by microscopy (Fig. 1b, Supplementary Data 2). We identified 102 GRACE strains that filament robustly in the absence of an inducing cue following overnight growth in medium with DOX, suggesting that these genes encode repressors of filamentation (Supplementary Data 3). Included in this set are many known morphogenetic regulators, such as the mitotic cyclin Clb2 (ref. 13; Fig. 1b) and the cyclin-dependent kinase Cdc28 (ref. 14). Consistent with a role for the cell cycle in morphogenesis^15^, this set included 50 genes with Gene Ontology (GO) terms associated with cell cycle, many of which had not been previously implicated in morphogenesis, including members of the structural maintenance of chromosomes complex and regulators of DNA replication (Supplementary Data 4).

We also identified 91 strains that remained entirely as yeast in the presence of serum and DOX (Supplementary Data 5), along with 508 strains that exhibited more moderate filamentation defects (Supplementary Data 6). Included among the gene set important for filamentation are six genes with homologues of unknown function in the model yeast *S. cerevisiae* (*YMR010W*, *YNL181W*, *YOR338W*, *YJR111C*, *YLR352W* and *YML131W*), 38 genes that lack identifiable homologues in *S. cerevisiae* and 10 genes that are fungal specific. Of the fungal-specific genes, four (*ARO1, ARO7, ARO2* and *RIB1*) are essential, suggesting potential new targets for antifungal therapies.

We examined the relationship between morphology and virulence, leveraging a previous analysis of virulence of 177 of the GRACE strains^16^. Most strains defective in filamentation had attenuated virulence or were avirulent, while most strains competent for filamentation were fully virulent or had severe growth defects associated with reduced virulence (Supplementary Fig. 3).

We focused further effort on a subset of strains with strong defects in filamentation to assess growth rates and specificity of the morphological defect. By monitoring growth kinetics at high resolution, we identified only a few strains with major defects in growth upon DOX treatment, confirming that most filamentation defects cannot be attributed simply to inhibition of growth (Supplementary Fig. 2). To determine whether the filamentation defects are specific to serum, we monitored morphology of this set of strains in response to different filament-inducing cues. Most strains had consistent defects in filamentation under all liquid medium conditions tested, demonstrating the utility of serum as a representative filamentation-inducing cue. However, clustering analysis revealed distinct filamentation programmes between liquid and solid medium (Fig. 1c).

To complement the detailed phenotypic analysis of the mutant strains and provide a new platform for future parallel condition high-throughput quantitative morphological screens, we developed a pooled-strain approach^17^,^18^. As a proof-of-concept experiment, we mixed populations of yeast (expressing green fluorescent protein) and filaments (expressing red fluorescent protein), then successfully re-separated the populations by passage through a 10-μm mesh filter (Fig. 1d). Filaments were effectively captured by the filter while yeast largely passed through unhindered.

To apply this concept on a large scale, we exploited the unique barcode sequences that mark 1,481 GRACE strains and all of the HET deletion mutants. Replicate pools of serum-exposed GRACE strains grown in the absence or presence of 0.5 μg ml^−1^ DOX were subjected to the filtration assay followed by barcode sequencing of the pre-filtration, filter-retained and flow-through fractions. DOX-treated strains with a flow-through:filter-retained log_2_ ratio ≥4 median absolute deviations (MAD) were scored as defective in filamentation (Fig. 1e, Supplementary Fig. 4). Of the 270 functionally barcoded strains with defects in serum-induced filamentation identified in the arrayed screen, 115 were also identified in the pooled assay (Fig. 1f). For strains that are completely unable to filament, the pooled assay demonstrated very high accuracy, identifying 19 of the 19 pertinent strains from the arrayed screen. An additional 73 mutants showed quantitative filamentation defects in the pooled screen that were not discovered in the initial arrayed analysis, in part due to the qualitative nature of microscopy. Thus, pooled screens with the GRACE collection provide a powerful, high-throughput approach to quantitatively assess cellular morphology.

To identify genes whose reduced dosage influences morphogenesis on a genome scale, we performed pooled morphology screens with the 5,296 HET deletion mutants in response to 10% serum (Supplementary Data 7). This expands on previous large-scale haploinsufficiency screens of heterozygous transposon insertion mutant collections, which identified 146 genes that influence filamentation on Spider and serum agar medium^19^. Using high-throughput screening, we identified 226 genes potentially demonstrating haploinsufficiency for morphogenesis (Fig. 1g, Supplementary Fig. 4), including those encoding known morphogenetic regulators such as *NRG1*, *TUP1* and *TEC1* (ref. 8), and genes reported to be haploinsufficient for filamentation based on a previous transposon screen such as *SPT5*, *SPT6* and *CDC39* (ref. 19). We confirmed hypofilamentous and hyperfilamentous phenotypes for a subset of heterozygous deletion strains by individual microscopy (Supplementary Fig. 5), although consistent with other studies^19^, morphological phenotypes of the heterozygous mutants were generally subtle.

Collectively, our screens identified 872 mutants with varying degree of defect in filamentation in response to serum. GO analysis was performed using GO Term Finder tools available at the *Candida* Genome database (www.candidagenome.org); this search revealed significant enrichment (*P*<0.05, hypergeometric distribution with Bonferroni correction) for genes involved in vesicle and intracellular protein transport, which are known to be required for polarized growth (Supplementary Data 4). Intriguingly, we observed that GO terms for ergosterol biosynthesis and N-linked glycosylation were also enriched (Supplementary Data 4). Although filamentous growth was not a significantly enriched GO term, likely because the GRACE collection targets ~1,800 genes that have not been previously examined for filamentation, many known key morphogenetic regulators were detected, including Ras1, Hsp90, Cdc24, Cyr1 and Flo8. Collectively, the results validate our screening approaches and highlight novel morphogenetic regulators.

Direct comparison of our screens with previous large-scale analyses of morphogenesis performed by Noble *et al.*^4^ and Homann *et al.*^11^ was hampered by differences in experimental conditions (Supplementary Fig. 6a). Comparison of mutants represented in the GRACE collection with those present in the aforementioned libraries and screened in response to serum in liquid medium by Ryan *et al.*^10^ revealed limited overlap of morphogenetic regulators (Supplementary Fig. 6b), possibly suggesting that differences in the nature of the genetic perturbation may influence morphological consequences. Notably, homozygous deletion of genes important for growth can result in triplications or suppressor mutations. This underscores the power of the GRACE collection where phenotypes can be specifically attributed to transcriptional repression of the target gene.

Our large-scale analysis of morphogenesis allowed us to examine the extent of conservation or divergence of morphogenetic circuitry between *C. albicans* and *S. cerevisiae*. We compared our set of genes influencing morphogenesis with those required for *S. cerevisiae* biofilm, haploid invasion and diploid pseudohyphal growth programmes^10^. Similar to the divergence in essentiality, we observed distinct sets of genes required for filamentation between the two species (Fig. 1h). Only genes required for biofilm growth were significantly correlated with morphogenetic regulators in *C. albicans* (*P*<0.01, Fisher’s exact test), and the positive predictive value was only 32.6%. This finding underscores the importance of elucidating regulatory circuitry governing morphogenesis directly in the pathogen.

### Ergosterol biosynthesis and morphology

**Figure 2:**
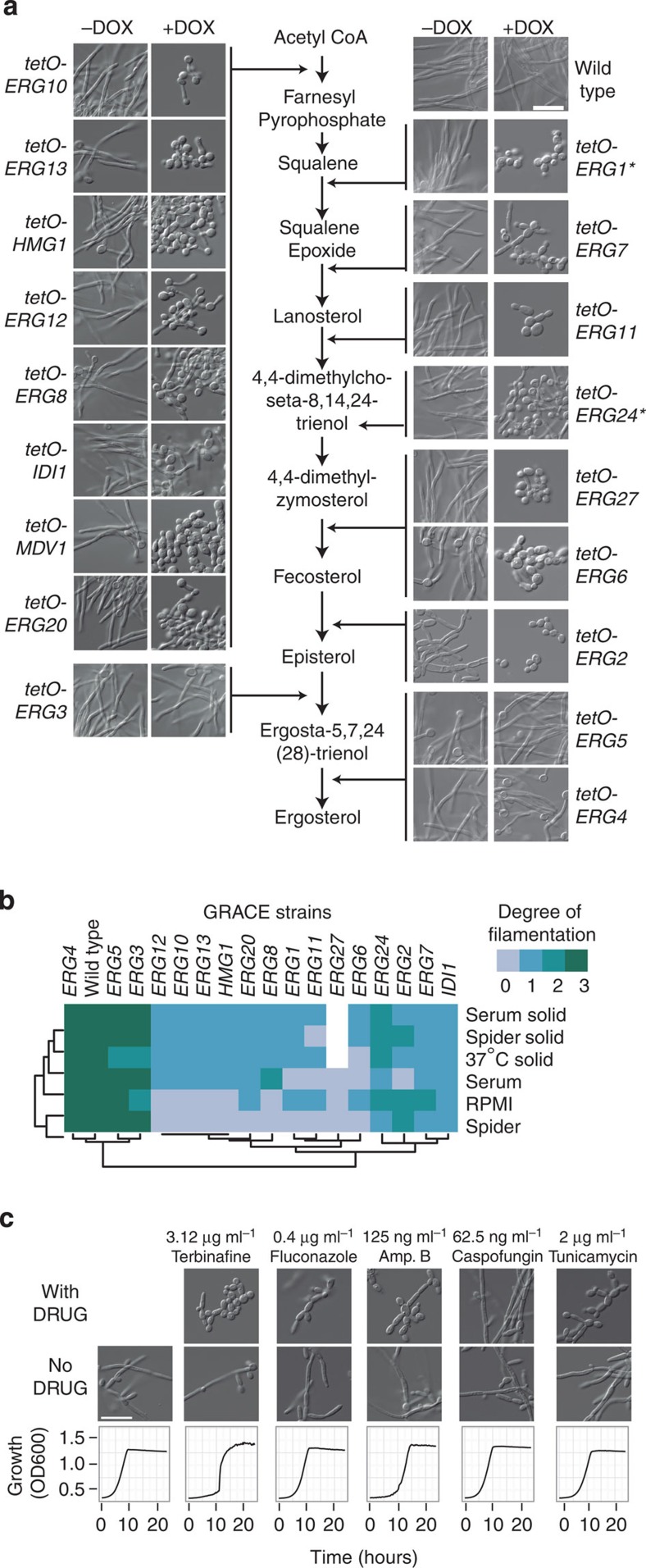
Ergosterol biosynthesis is important for filamentation. (**a**) Transcriptional repression of most ergosterol biosynthesis genes with 0.05 μg ml^−1^ DOX blocks filamentation induced by serum. An asterisk indicates strains where 1 μg ml^−1^ DOX was used to repress target gene transcription. For all images: Scale bar, 20 μm. Ergosterol biosynthetic pathway adapted from *S. cerevisiae*^20^. (**b**) Clustering analysis of GRACE strains for ergosterol biosynthesis genes and phenotypes observed in distinct filament-inducing conditions with 0.05 μg ml^−1^ DOX. White boxes indicate a lack of growth in that condition. Clustering was performed using the Heatmap function in R. (**c**) Treatment with sub-lethal concentrations of specific antifungal drugs reversibly blocks filamentation induced by serum. Top panels: morphology in the presence of drug. Middle panels: morphology after removal of drug. Bottom panels: growth kinetics analysis demonstrates that the drugs do not significantly inhibit growth at the concentrations used.

We observed that the ergosterol biosynthesis pathway was enriched for GRACE strains defective in filamentation. We further examined the phenotypes of 18 GRACE strains whose open reading frames (ORFs) had significant homology to *S. cerevisiae* genes involved in ergosterol biosynthesis^20^ (Fig. 2a). Transcriptional repression of all genes involved in the early stages of ergosterol production, up to the biosynthesis of episterol, resulted in defects in filamentation, while repression of genes encoding enzymes involved in the conversion of episterol to ergosterol did not. The defects in filamentation were not specific to serum, but were common to multiple inducing cues (Fig. 2b).

We took a complementary pharmacological approach to confirm the importance of ergosterol biosynthesis in filamentation^9^. Treatment with fluconazole (which targets Erg11), terbinafine (which targets Erg1) or amphotericin B (which binds ergosterol) blocks filamentation induced by serum (Fig. 2c). These effects are observed at concentrations where there is no growth inhibition and are reversed on removal of the antifungal drugs. Similar treatment with caspofungin, which disrupts cell-wall integrity, does not affect filamentation. While some ergosterol biosynthesis genes have been previously implicated in filamentation^16^,^21^,^22^, this is the first comprehensive analysis and provides insight into the relationship between pathway structure and morphogenesis.

### N-linked glycosylation and morphology

**Figure 3:**
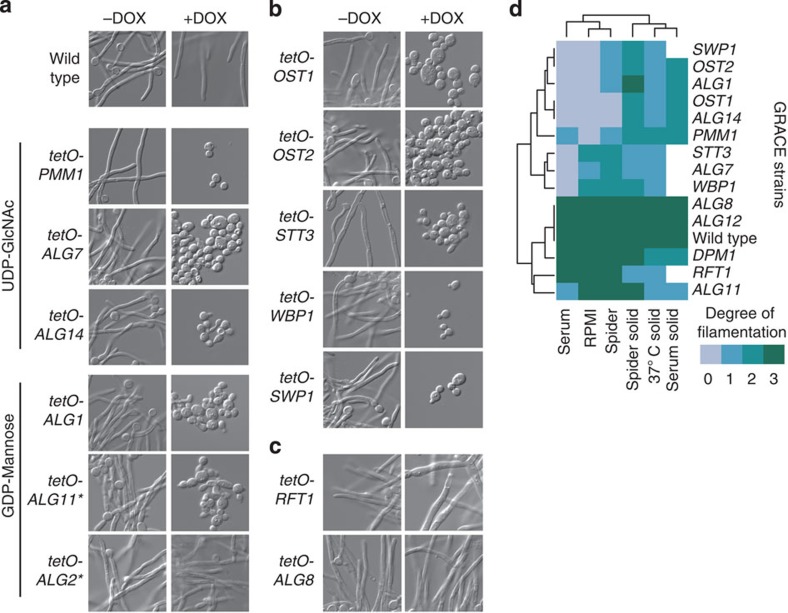
Glycosylation genes influence filamentation. (**a**) Depletion of cytosol-oriented glycosyltransferases with 0.05 μg ml^−1^ DOX blocks filamentation induced by serum. An asterisk indicates strains where 1 μg ml^−1^ DOX was used to repress target gene transcription. For all images: scale bar, 20 μm. (**b**) Depletion of the oligosaccharyltransferase complex blocks filamentation induced by serum. (**c**) Depletion of lumen-oriented glycosyltransferases does not affect filamentation. (**d**) Clustering analysis of GRACE strains for N-linked glycosylation genes and phenotypes observed in distinct filament-inducing conditions with 0.05 μg ml^−1^ DOX. White boxes indicate a lack of growth in that condition.

The biological pathway responsible for N-linked glycosylation also emerged as fundamental to serum-induced filamentation. We determined that the glycosyltransferases on the cytoplasmic side of the endoplasmic reticulum, which are responsible for attachment of N-acetylglucosamine or mannose to dolichol pyrophosphate^23^, are required for filamentation induced by serum (Fig. 3a). We confirmed this observation pharmacologically by treating wild-type cells with sub-inhibitory concentrations of tunicamycin, a nucleoside antibiotic that targets Alg7 to block N-linked glycosylation, and noted a reversible block of filamentation (Fig. 2c). The enzymes of the oligosaccharyltransferase complex, which are responsible for transfer of glycan to polypeptide chains^23^, were also required for filamentation induced by serum (Fig. 3b). By contrast, the lumen-oriented glycosyltransferases, which extend the branched mannose chains on the glycan, were not required for filamentation (Fig. 3c). This pattern in filamentation defects was not specific to serum, but was observed in response to multiple inducing cues (Fig. 3d). Strikingly, the GRACE strain targeting the *C. albicans* homologue of *RFT1* has enhanced filamentation in response to serum (Fig. 3c). In *S. cerevisiae,* Rft1 is involved in translocating the oligosaccharide to the lumen of the ER, and depletion of Rft1 phenocopies depletion of the cytosol-oriented glycosylation proteins^24^. Together, these finding implicate the N-linked glycosylation pathway in regulating filamentation and illuminates divergence of essential gene function between *C. albicans* and *S. cerevisiae*.

### Cell surface moieties induce pyroptosis

**Figure 4:**
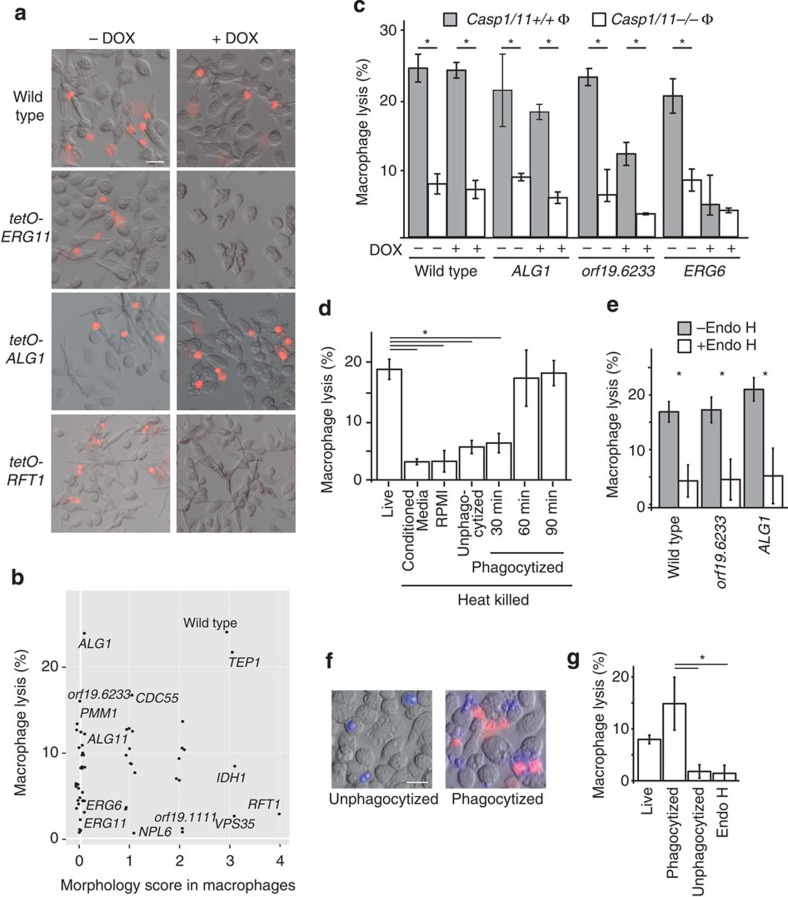
Elucidating the relationship between *C. albicans* morphology and macrophage lysis. (**a**) Representative images of infected macrophages stained with propidium iodide to identify dead cells (in red). Images were taken 4 h post inoculation. (**b**) *C. albicans* morphogenesis is uncoupled from macrophage lysis. Macrophages were inoculated with GRACE strains in the presence of 0.05 μg ml^−1^ DOX; lysis events and *C. albicans* morphogenesis were imaged 4 h post infection. (**c**) Lysis is due to caspase-1-dependent pyroptosis. Wild-type or *casp1*^*−/−*^ *casp11*^*−/−*^ macrophages were infected with the indicated GRACE strains in the presence or absence of 0.05 μg ml^−1^ DOX. Lysis events were imaged 4 h post inoculation. (**d**) Wild-type *C. albicans* cells heat killed following prior internalization by macrophages can drive macrophage lysis. Wild-type *C. albicans* cells were treated with the indicated conditions before being heat killed and used for infection of J774A.1 macrophages. Lysis events were imaged 4 h post inoculation. (**e**) Specific GRACE strains can drive macrophage lysis following heat killing after prior macrophage phagocytosis, and this process is dependent on mannosylated proteins. The indicated DOX-grown strains were used to infect macrophages, collected after 90 min, washed and heat killed. The cells were treated with Endo H overnight, washed and used for inoculation of J774A.1 macrophages. (**f**) Heat-killed acapsular *C. neoformans* cells can induce macrophage lysis after pre-exposure to the host. *cap59Δ* mutant cells were incubated within macrophages for 4 h, collected, washed and heat killed. The cells were then used for inoculation of J774A.1 macrophages. Infected macrophages were stained with propidium iodide to identify dead cells (in red) and with calcofluor white to identify fungal cells (in blue). Lysis events were imaged 4 h post infection. (**g**) The ability of *C. neoformans* to induce pyroptosis is dependent on mannosylated proteins. *cap59Δ* cells were treated with the indicated conditions before being used for infection of J774A.1 macrophages. For all graphs, data are represented as mean±s.d. for triplicate samples, *n*=200 infection events, asterisk indicates *P*<0.01, unpaired *t*-test. For all images, scale bar, 20 μm.

*C. albicans* yeast and filamentous cells differ in physiological, structural and biochemical features that modulate immune recognition and survival in macrophages, thereby shaping the resulting host immune response^12^. Although macrophages can kill some internalized *C. albicans* yeast cells, the majority survive and filament in response to engulfment by the phagosome, with filamentation temporally coupled to induction of macrophage pyroptosis, a highly inflammatory cell death programme that is activated via caspase-1 (ref. 25). The current paradigm is that filaments are necessary but not sufficient to induce macrophage lysis, and the factors driving lysis remain largely enigmatic^25^,^26^,^27^. Therefore, we examined the ability of our filament-blocked strains to induce this process. We inoculated J774A.1 murine macrophages with *C. albicans* cells and used propidium iodide (PI) staining to identify lysed macrophages^27^. Four hours post inoculation, we used imaging to quantify lysed macrophages and assess *C. albicans* morphology during infection (Fig. 4a). As expected, most of the filament-blocked GRACE strains were unable to induce lysis (Fig. 4b, Supplementary Table 1). Consistent with previous reports^26^, some mutants were unable to induce macrophage lysis despite having significant hyphal formation; for example, the *RFT1-* or *VPS35-*depleted cells had extensive filaments within macrophages but did not induce lysis (Fig. 4a,b). Strikingly, by contrast, the *ALG1-, ALG11-* and *orf19.6233*-depleted GRACE strains were still able to induce macrophage lysis despite being blocked in filamentation (Fig. 4b). Infection of *casp1*^*−/−*^ *casp11*^*−/−*^ macrophages with these mutant strains revealed a significant reduction in macrophage lysis rates (Fig. 4c), demonstrating that the observed lysis is due to caspase-1-dependent pyroptosis.

To investigate the factors required for inducing macrophage lysis, we examined the capacity of our wild-type strain to induce lysis after incubation under different conditions. Consistent with previous results^26^, rich medium grown heat-killed wild-type cells were unable to trigger macrophage lysis (Supplementary Table 2). Pre-incubation of wild-type cells in RPMI or macrophage-conditioned medium for 2 h prior to heat killing was also ineffective at inducing macrophage lysis (Fig. 4d). Finally, we examined the effect of heat-killed wild-type cells that had been previously phagocytized by macrophages. Killed *C. albicans* cells previously phagocytized for 60 or 90 min induced robust macrophage lysis, despite being yeast form at these time points (Fig. 4d). Similar results were seen with previously phagocytized formalin-killed cells (Supplementary Table 2). The ability to induce lysis was contingent on ingestion by macrophages; heat-killed *C. albicans* cells that had been co-incubated with macrophages but not phagocytized were unable to induce lysis (Fig. 4d). Addition of heat-killed macrophages had no effect on lysis rates. Moreover, conditioned medium from the co-incubation was also unable to induce lysis, suggesting that lysis is not driven by a secreted factor.

The capacity of heat-killed previously phagocytized cells to induce macrophage lysis was shared by the filament-defective *ALG1-* and *orf19.6233-*depleted GRACE strains (Supplementary Table 2, Fig. 4e). However, not all strains were able to induce macrophage lysis in these conditions. For example, *ERG6-* and *OST1-*depleted yeast cells have a defect in macrophage lysis that is unaffected by killing after macrophage internalization (Supplementary Table 2). Together, these observations suggest that *C. albicans* remodels its cell surface in response to macrophage phagocytosis, exposing moieties capable of inducing macrophage lysis. Mutants that are unable to remodel their cell surfaces in response to phagocytosis are therefore not able to induce macrophage lysis.

Glycosylated cell-wall mannoproteins are major contributors to innate immune recognition^28^. The exposure of cell-wall mannans is a highly dynamic process subject to influence by environmental conditions^28^. To determine if glycoproteins are required for inducing lysis, we treated heat killed, previously phagocytized wild-type, *orf19.6233* and *ALG1-*depleted cells with Endo H glycosidase to remove glycoproteins from the cell surface. These Endo H-treated cells were unable to induce macrophage lysis, suggesting that highly mannosylated cell surface proteins are necessary for triggering the host response (Fig. 4e).

To assess whether the capacity of dead, previously phagocytized yeast cells to activate macrophage lysis is specific to *C. albicans* or is more broadly conserved across fungi, we turned to the evolutionarily distant pathogen *C. neoformans*. An acapsular strain of *C. neoformans* (*cap59Δ*) can induce activation of macrophage inflammatory pathways in a manner thought to be contingent on fungal cell viability^29^. Consistent with an anti-inflammatory and anti-phagocytic role for capsule^30^, a heat-killed encapsulated wild-type strain did not induce macrophage lysis, even after pre-ingestion by macrophages (Supplementary Table 2). However, the heat-killed acapsular *cap59*Δ mutant induced robust lysis if previously phagocytized by macrophages (Fig. 4f). Again, this ability to induce lysis was contingent on glycosylated mannoproteins since Endo H treatment decreased lysis rates (Fig. 4g). We also tested previously phagocytized and heat-killed *S. cerevisiae* (S288C), and found that these cells do not induce macrophage lysis, suggesting that cell-wall remodelling to induce macrophage lysis is a pathogen-specific trait (Supplementary Table 2). Together, these observations suggest that fungal pathogens actively remodel their cell surface in response to the phagosome environment, thereby exposing glycosylated proteins that trigger macrophage lysis, and that this process can be uncoupled from filamentation.

## Discussion

The ability to transition between morphotypes is a key virulence trait for diverse fungal pathogens^1^. Here we analysed morphogenesis in the human fungal pathogen *C. albicans,* using arrayed phenotyping and massively parallel pooled screening to assess filamentation in response to a variety of inducing cues. This analysis expands our appreciation of morphogenetic circuitry beyond previously known processes that influence filamentation, including vesicle and intracellular protein transport and the Ras signalling cascade^8^,^15^,^31^. We found that the majority of genes encoding repressors of filamentation are linked to cell cycle progression, suggesting that polarized growth is a ubiquitous consequence of cell cycle perturbation in *C. albicans*. In addition, the parallel genome-scale approaches of our analyses identified hundreds of novel morphogenetic regulators, including many fungal-specific factors and uncharacterized genes. The morphology profiles provide a rich phenotypic signature of gene function, as has been achieved with systematic analysis of morphology in *S. cerevisiae*^32^,^33^.

Most antifungals in clinical use target ergosterol or its biosynthesis^9^, and can have a profound impact on cellular biology and signalling mediated through changes in cell membrane composition or ergosterol-protein interactions that influence protein function^34^. An example are the azoles, which inhibit ergosterol biosynthesis and block filamentation^18^. Azoles’ effect on cellular morphology is thought to be caused by either increased levels of the quorum sensing molecule farnesol, or by prevention of ergosterol localization to the tip of the polarizing cell^9^,^35^. Our systematic analysis of filamentation phenotypes on perturbation of ergosterol biosynthesis at each step of the pathway reveals the importance of the early and middle stages of ergosterol biosynthesis, beyond conversion of farnesyl pyrophosphate to other intermediates. This suggests that farnesol accumulation is unlikely to be the mechanism by which azoles impair filamentation, a conclusion that is further supported by our finding that all of the barcoded ergosterol biosynthesis mutants had defects in filamentation in the pooled analysis, where the vast majority of cells present in the pool would be competent for farnesol production. Moreover, transcriptional repression of the late genes that convert episterol to ergosterol does not block filamentation, suggesting that decreases in membrane ergosterol levels are not sufficient to explain the morphogenetic defects. Instead, we hypothesize that the filamentation defects are likely attributable to the accumulation of specific sterol intermediates. The impact of antifungals on filamentation may also modulate interactions with host immune cells, with therapeutic implications.

As a ubiquitous commensal and opportunistic pathogen, *C. albicans* has evolved complex interactions with host immune cells such as macrophages. *C. albicans* filamentation induces macrophage pyroptosis, a highly inflammatory active cell death programme, via the NLRP3 inflammasome and caspase-1 activation^25^,^36^. Mice lacking components of the NLRP3 inflammasome have increased fungal burden and decreased survival, potentially due to an inability to recruit neutrophils and shape an appropriate immune response^36^. To date, the standard paradigm has been that filamentation is required for pyroptosis, although the discovery of mutants that are able to filament in macrophages without causing lysis suggests that filamentation is not sufficient for lysis^26^,^27^. Here we demonstrate that transcriptional repression of *ALG1*, *ALG7* or *orf19*.*6233* causes defects in filamentation but does not block macrophage lysis, providing compelling evidence that filamentation is not a prerequisite for lysis.

Instead, we propose that the capacity of *C. albicans* to induce macrophage pyroptosis is mediated by remodelling of the cell surface on ingestion by host macrophages. We discovered that wild-type *C. albicans* actively remodels its cell surface in response to the phagosome environment, and this altered cell surface is sufficient to induce macrophage lysis in the absence of *C. albicans* growth. Heat-killed *ALG1-* or *orf19.6233-*repressed cells are also able to induce lysis after previous phagocytosis by macrophages, demonstrating that this cell surface alteration does not depend on filamentation. However, the process is likely to be coordinately regulated^37^, as many filament-defective strains remained unable to induce pyroptosis even after pre-exposure to macrophages. Recent work has implicated O-linked glycosylation in phagosome maturation and escape of live *C. albicans* cells from phagocytes^38^. We observed that treatment with the glycosidase Endo H abolished the ability of wild-type cells to induce lysis, consistent with a role for mannoproteins in regulating interactions with the host^12^,^28^. The capacity of killed fungal cells to drive macrophage lysis following cell-wall remodelling in response to phagosome conditions was also observed with *C. neoformans*, suggesting a potentially broad relevance for host–pathogen interactions. Host recognition of fungal pathogens involves receptor interactions with multiple components of the fungal cell surface, and coordinated stimulation of the phagocyte by numerous antigens is important in shaping immune responses^12^. Mechanisms to alternately mask and reveal immunostimulatory molecules pervade biology, resulting in the stunning complexity of host–pathogen interactions that have emerged over evolutionary time^12^,^30^,^39^.

Targeting pathogen virulence traits required for causing infection in the host may be an attractive strategy for drug development in this era of antimicrobial resistance. The efficacy of targeting virulence factors has been established in many bacterial species, including inhibitors of the type III secretion systems, which prevent translocation of effector molecules and are effective at attenuating *Yersinia pseudotuberculosis* infections and inhibiting replication of *Chlamydia trachomatis* and *Chlamydophila pneumoniae ex vivo*^40^. Targeting virulence traits offers numerous benefits, including expanding the repertoire of antifungal targets, minimizing effects on the host endogenous microbiome and reducing selective pressure for the evolution of drug resistance.

## Methods

### Growth conditions

*C. albicans, C. neoformans* and *S. cerevisiae* strains were grown in standard conditions at 30 °C in YEPD (2% peptone, 2% dextrose and 1% yeast extract). Depletion of *C. albicans* target gene expression was achieved by adding the indicated concentrations of DOX to the growth medium. *C. albicans* strains were created by Merck and Genome Canada and distributed by the National Research Council. The *C. neoformans* wild-type (H99) and acapsular (*cap59Δ)* strains were a gift from John Perfect (Duke University) and the *S. cerevisiae* strain S288C was from Susan Lindquist (Whitehead Institute). Archives of all strains were maintained in 25% glycerol at −80 °C.

### Essentiality screening

Essentiality was determined as previously described^7^. During strain construction, each strain was struck onto YNB (0.17% yeast nitrogen base without amino acids without ammonium sulfate, 2% dextrose, 0.1% glutamic acid and 2% agar)- and growth in the absence of tetracycline was compared with growth in the presence of 100 μg ml^−1^ tetracycline after 48 h at 30 °C. The essential genes were identified using an independent method of streaking strains onto YNB containing 2% glucose, 1,000 μg ml^−1^ 5-fluoroorotic acid and 100 μg ml^−1^ uridine to select for ura- cells which have excised the transactivator, removing transcription of the target gene. Essentiality of each strain in the NRC-distributed GRACE collection was verified by comparing the colony size of each strain transferred via 96-well replicator (Singer Instruments) from overnight cultures to YEPD agar plates in the absence or presence of 1 μg ml^−1^ DOX and grown at 30 °C for 48 h. Additional analysis comparing colony sizes in the presence or absence of 100 μg ml^−1^ DOX on YNB agar plates was performed for confirmation when necessary. For *S. cerevisiae* essentiality, phenotypes were obtained from the Saccharomyces Genome Database (yeastgenome.org). Homologues between *S. cerevisiae* and *C. albicans* were identified from the *Candida* Genome Database.

### Reverse transcription–PCR

To monitor transcriptional gene repression of the GRACE strains upon treatment with DOX, strains were grown overnight in YEPD at 30 °C in the presence or absence of 0.05 μg ml^−1^ DOX, diluted to OD600 of 0.1 in the same conditions and grown to mid-log phase. Cultures were pelleted and frozen at −80 °C.

RNA was isolated using the QIAGEN RNeasy kit and RNasefree DNase (QIAGEN). Complementary DNA synthesis was performed using the AffinityScript Multi Temperature cDNA Synthesis Kit (Agilent Technologies). PCR was performed using FastSYBR Green Master Mix (Applied Biosystems) and BioRad CFX384 Real Time System, with the following cycling conditions: 95 °C for 3 min, then 95 °C for 10 s and 60 °C for 30 s, for 40 cycles. Reactions were performed in triplicate, for two biological replicates using the primers oLC1131/oLC1132 (*ERG11*), oLC2285/oLC2286 (*ACT1*), oLC3546/oLC3547 (*PMM1*), oLC3550/oLC3551 (*ALG7),* oLC3552/oLC3553 (*ERG6)* and oLC3554/oLC3555 (*ERG20)*. Primer sequences are included in Supplementary Table 3. Data were analysed using the BioRad CFX Manager 3.1. All data were normalized to the *ACT1* reference gene. Error bars show the s.e.

### Arrayed morphology screens

The GRACE library was screened by microscopy. Overnight cultures were grown in in 100 μl YEPD. in 96-well plates at 30 °C under static conditions. The cells were then transferred via 96-well replicator into either rich medium (YEPD) or rich medium containing 0.05 μg ml^−1^ DOX (YEPD DOX) and incubated at 30 °C for 18 h under static conditions. The strains were then inoculated into media with specific filamentation-inducing cues in a 96-well format and incubated; 4 h at 37 °C for serum YEPD with 10% v/v heat-inactivated newborn calf serum (Gibco #26010-066), 5 h at 37 °C for RPMI medium (Gibco #11875-093) and 6 h at 30 °C for Spider medium (1% mannitol, 1% nutrient broth and 0.2% K_2_HPO_4_). Images of each well were captured on a Zeiss Axio Observer.Z1 (Carl Zeiss) using × 40 magnification. The strains were scored for degree of filamentation on a scale from 0 (unable to filament), 1 (very short or pseudohyphae), 2 (short) or 3 (fully filamentous) (Supplementary Fig. 7). To validate phenotypes, individual strain imaging from liquid media was performed after growth in static conditions in the inducing conditions above using differential interference contrast microscopy on a Zeiss Axio Imager.MI at × 40 magnification.

### Solid media morphology screens

Mutant strains were grown overnight in YEPD in 96-well plates under static conditions at 30 °C and transferred via 96-well replicator onto the indicated agar plates (2% agar) with or without 0.5 μg ml^−1^ DOX. The plates were incubated for 3 days at either 30 °C (for Spider medium) or 37 °C (for serum medium) before imaging. Each strain was scored on a scale from 0 to 3 for degree of filamentation and colony morphology. Examples of these scores are shown in Supplementary Fig. 7.

### Growth kinetics

To assess the growth kinetics on repression of gene transcription, GRACE strains were grown overnight in YEPD in 96-well format under static conditions at 30 °C and transferred via 96-well replicator into 100 μl of YEPD in the presence or absence of 0.05–0.5 μg ml^−1^ DOX. The strains were grown in a TECAN GENios plate reader and incubator at 37 °C, with orbital shaking on high. Absorbance readings were measured at 595 nm every 15 min for 24 h, using the XFluor4 software. Growth kinetics graphs are an average of two biological replicates, with three technical replicates each.

### Drug assays

To assay the growth kinetics on drug treatment, the wild-type strain was transferred via 96-well replicator from an overnight culture into 100 μl of YEPD with the indicated concentration of fluconazole (Sequoia Research Products Ltd SRP01025f), terbinafine (Sigma T8826), amphotericin B (Sigma A4888), caspofungin (Merck) or tunicamycin (Sigma T7765). TECAN analysis was performed as above. Growth kinetics graphs are an average of two biological replicates, with three technical replicates each.

To assess the effect of the drugs on filamentation, the wild-type strain was transferred via 96-well replicator from an overnight culture into 100 μl of YEPD with the indicated amount of drugs overnight at 37 °C, and then pinned into 100 μl of YEPD with 10% serum in the absence or presence of the drug. Images of the cells were taken after 4 h at 37 °C.

### GRACE and HET pool growth

Barcoded *C. albicans* GRACE glycerol stock pools were thawed, diluted into replicate 50 ml YEPD cultures to an OD600nm of 0.05 and grown at 30 °C with shaking. DOX was added to one culture (0.5 μg ml^−1^ final concentration) after 90 min and both cultures were then incubated for a further 15-h period. The two cultures were each sub-cultured into triplicate 10 ml cultures with fresh medium at an OD600nm of 0.05 with or without 0.5 μg ml^−1^ DOX and incubated at 30 °C with shaking for 180 min. Bovine calf serum was then added (10% v/v) to all six cultures and the incubation temperature was shifted to 37 °C until cultures demonstrated robust early filamentation (~160 min).

Barcoded *C. albicans* HET glycerol stock pools were thawed, diluted into triplicate 10 ml YEPD cultures to OD600 of 0.05 and grown at 30 °C with shaking for 180 min. Bovine calf serum was then added (10% v/v) to all three cultures and incubation temperature was shifted to 37 °C until cultures demonstrated robust early filamentation (~160 min).

### Filtration

Prior to filtration, 1 ml aliquots of each sample were collected. For each sample, remaining culture was filtered at a rate of ~1 ml s^−1^ through a 10-μm mesh nylon membrane (Small Parts #CMN-0010-D) sandwiched in a microfiltration apparatus (VWR#71001-592) inserted into the mouth of a 1 l side-arm flask under mild vacuum. Membranes were washed with 35 ml of fresh YEPD medium. Cells adhered to the membrane surface were collected by scraping, and cells that passed through the membrane were collected by centrifugation of the flow-through fraction.

### Genomic DNA preparation and sequencing

Genomic DNA was prepared from cell pellets using the MasterPure yeast DNA purification kit (EpiCentre #MPY80010). DNA was quantified by the Quant-iTTM PicoGreen dsDNA assay kit (Life Technologies #7589). Barcode amplification by PCR was performed using 78 ng of genomic DNA for GRACE pool samples and 180 ng of genomic DNA for HET pool samples via the Takara Ex-Taq enzyme (Clontech #RR001) using the following thermal cycler program:

1. 94 °C, 120 s
2. 94 °C, 20 s
3. 53 °C, 20 s
4. 72 °C, 14 s
5. GoTo step 2, × 28
6. 72 °C, 1 min
7. 4 °C

UPTAG and DNTAG primer sequences are included in Supplementary Table 3. Separate UPTAG and DNTAG multiplexed pools were formed by combining equal amounts of PCR product from each sample. Pooled UPTAG and DNTAG DNA pools were electrophoresed on a 5% 1 × TBE polyacrylamide gel and recovered by eluting DNA from shredded gel slices in 10 mM Tris-HCl pH8.0. Equal quantities of UPTAG and DNTAG pools were combined to form a library, which was sequenced on an Illumina Hi-Seq 2500 instrument (single-end flow cell) using specific primers to sequence and index the UPTAG and DNTAGs (Supplementary Table 3).

Barcode sequence reads were mapped to an artificial genome containing known UPTAG and DNTAG sequences via Bowtie v1.0 (http://bowtie-bio.sourceforge.net/index.shtml). Read frequency for the UPTAG and DNTAG of each strain were compiled for each indexed sample. Correlation of the UPTAG and DNTAG reads are demonstrated in Supplementary Fig. 3. Experiments were performed with two biological replicates, with three technical replicates each.

Strains where pre-filtration read counts for both UPTAG and DNTAG were <1 read per million mapped reads were omitted from further analysis. Log_2_ fold difference for strain reads between filter fractions was calculated by averaging the log_2_ fold change for the UPTAG and DNTAG. If either barcode in a given strain had <1 read per million mapped reads, only the reads for the complementary barcode in a strain were used to calculate log_2_ fold difference. Strains were considered significantly enriched if the log_2_ flow-through:filter-retained ratio was ≥4 MADs. MADs were used instead of s.d. to minimize the effect of outliers. We chose 4 MAD as a stringent cutoff, with high precision and recall rates. For the HET library, 4 MAD=0.531; for the GRACE library with DOX, 4 MAD=0.705; and for the GRACE library without DOX, 4 MAD=0.527.

### Macrophage lysis

J774A.1 (ATCC) or caspase-1^−/−^ 11^−/−^ macrophages (gift from Stuart Levitz and Kate Fitzgerald) were counted using a haemocytometer, diluted and plated in a 24-well tissue culture dish at a final density of 5 × 10^5^ cells per well in RPMI-1640 medium (Gibco) containing 3% heat-inactivated FBS (Gibco). The cells were incubated at 37 °C with 5% CO_2_ for 18 h to activate the macrophages. Similar results were obtained when macrophages were activated for 1 h with 50 ng ml^−1^ lipopolysaccharide. GRACE strains were incubated for 18 h in YEPD with or without 0.05 μg ml^−1^ DOX (unless otherwise indicated) to deplete target gene expression. The cells were diluted to 1 × 10^6^ cells ml^−1^ in RPMI (±DOX), and 1 ml was added to each well of macrophages.

After 1 h of co-incubation, unphagocytized *Candida* cells were removed by washing × 3 with phosphate-buffered saline (PBS), and the medium was replaced with RPMI containing 1 μg ml^−1^ PI (Sigma-Aldrich), with or without DOX. Four hours post inoculation, five independent fields for each well were captured on a Zeiss Axio Observer.Z1 at × 10 magnification. PI staining was observed using an X-Cite series 120 light source and an ET HQ tetramethylrhodamine isothiocyanate (TRITC)/DsRED filter set. The per cent of infected macrophages demonstrating PI staining was determined using ImageJ. At least 200 infected macrophages were counted per strain. Each experiment was performed in triplicate, with two biological replicates. Statistical significance (*P*<0.05) was determined by unpaired *t*-tests (GraphPad). For *C. neoformans* and *S. cerevisiae* infections, the cells were stained with calcofluor white (0.1 mg ml^−1^) before imaging to aid in identification of fungal cells.

### Cell surface induction

J774A.1 cells were counted using a haemocytometer, diluted and plated in a six-well tissue culture dish at a final density of 1 × 10^5^ cells ml^−1^ in RPMI-1640 medium containing 3% heat-inactivated FBS. The cells were incubated at 37 °C with 5% CO_2_ for 18 h. Fungal cells were incubated overnight in YEPD (with or without 0.05 μg ml^−1^ DOX as indicated), diluted to an OD600 of 0.1 and added to the macrophages. *C. albicans* was incubated with macrophages for 60 or 90 min. *S. cerevisiae* was incubated with macrophages for 90 min. *C. neoformans* was incubated with macrophages for 4 h. After the indicated time points, the unphagocytized fungal cells were collected by gentle washing, and the phagocytized fungal cells were collected by scraping and lysing macrophages. The macrophage debris was removed by washing, and the fungal cells were then heat killed at 56 °C for 30 min or formalin fixed in 1% formaldehyde overnight. These cells were then used for subsequent infection of macrophages.

### Glycosidase treatment

Heat-killed fungal cells were incubated with Endo H (NEB #P0702S) in the provided G5 buffer and incubated at 37 °C overnight to remove high mannose *N*-glycans from glycoproteins. Cells were then washed in PBS, resuspended in RPMI and used for infections.

## Additional information

**How to cite this article:** O’Meara, T. R. *et al.* Global analysis of fungal morphology exposes mechanisms of host cell escape. *Nat. Commun.* 6:6741 doi: 10.1038/ncomms7741 (2015).

## Supplementary Material

Supplementary Information Supplementary Figures 1-7, Supplementary Tables 1-3 and Supplementary References

Supplementary Data 1 Essentiality data

Supplementary Data 2 GRACE morphology scores

Supplementary Data 3 Repressors of filamentation

Supplementary Data 4 GO term analysis

Supplementary Data 5 GRACE mutants with strong defects in filamentation in response to serum

Supplementary Data 6 All GRACE mutants with defects in filamentation in response to serum

Supplementary Data 7 HET pooled morphology analysis
