# Supplementary material for: Global analysis of fungal morphology exposes mechanisms of host cell escape
Source: Nat Commun. 2015 Mar 31;6:6741. doi: 10.1038/ncomms7741 (PMC4382923; doi:10.1038/ncomms7741)
Supplement: Supplementary Information — Supplementary Figures 1-7, Supplementary Tables 1-3 and Supplementary References [file ncomms7741-s1.pdf]

Supplementary Information

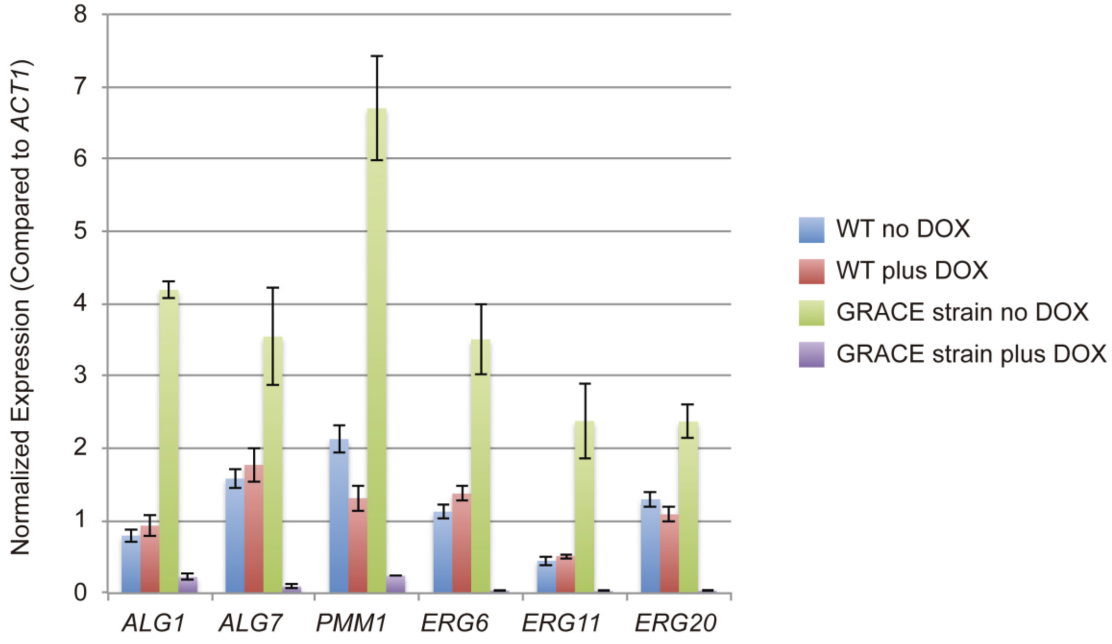

Supplementary Figure 1: Target gene transcript levels are depleted upon treatment with doxycycline (DOX). Strains were grown in the absence or presence of 0.05 µg mL^{-1} DOX overnight, subcultured into fresh medium of the same condition and grown to mid-log phase. Target gene transcript levels were normalized to *ACT1*. Data are means +/- S.D. for triplicate samples. Figure represents one of two biological replicates.

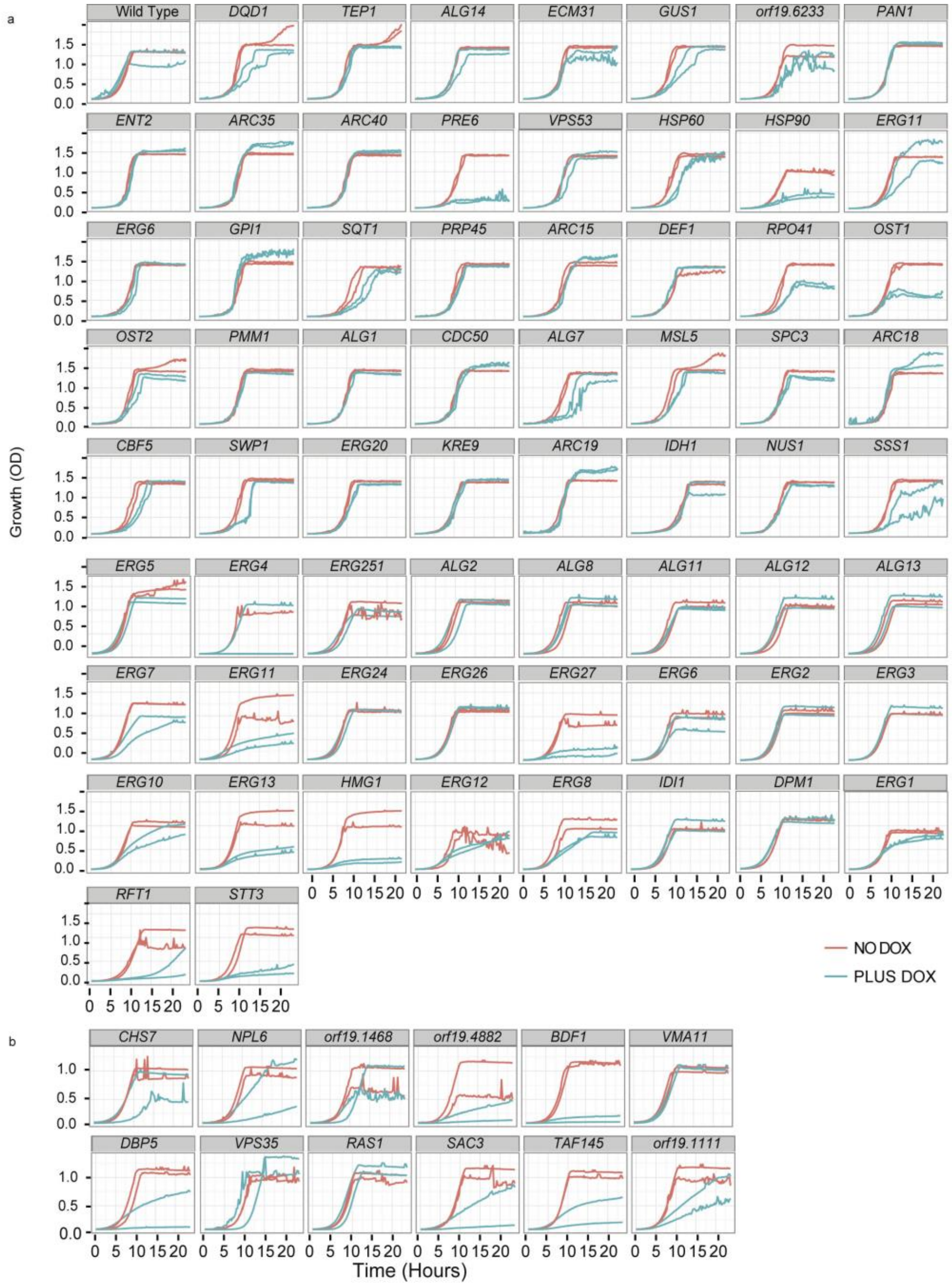

Supplementary Figure 2: Growth kinetics of GRACE strains. Strains were inoculated from overnight cultures into 96-well culture plates in the absence (in blue) and presence of DOX (in red) and incubated with shaking in a TECAN plate reader at 37°C. The OD600 values were measured every 15 minutes for 24 hours. Each graph displays two biological replicates performed in triplicate. **(A)** Strains grown in 0.05 μg mL^{-1} DOX. **(B)** Strains grown in 0.5 μg mL^{-1} DOX.

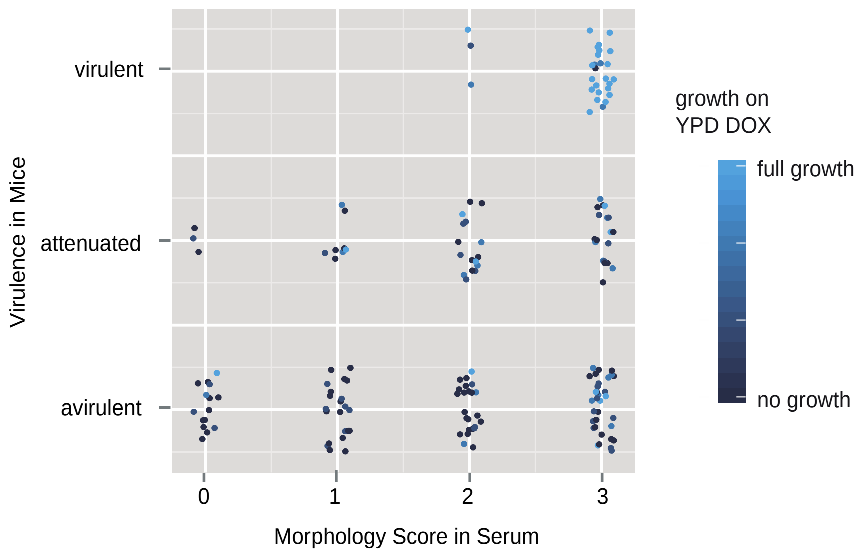

Supplementary Figure 3: Correlation between virulence in mice and morphology score in response to serum. Color bar indicates growth in rich medium. Morphology scores indicate the degree of filamentation, where 0 indicates yeast form and 3 indicates filaments. Virulence data from Becker *et al.* [1]

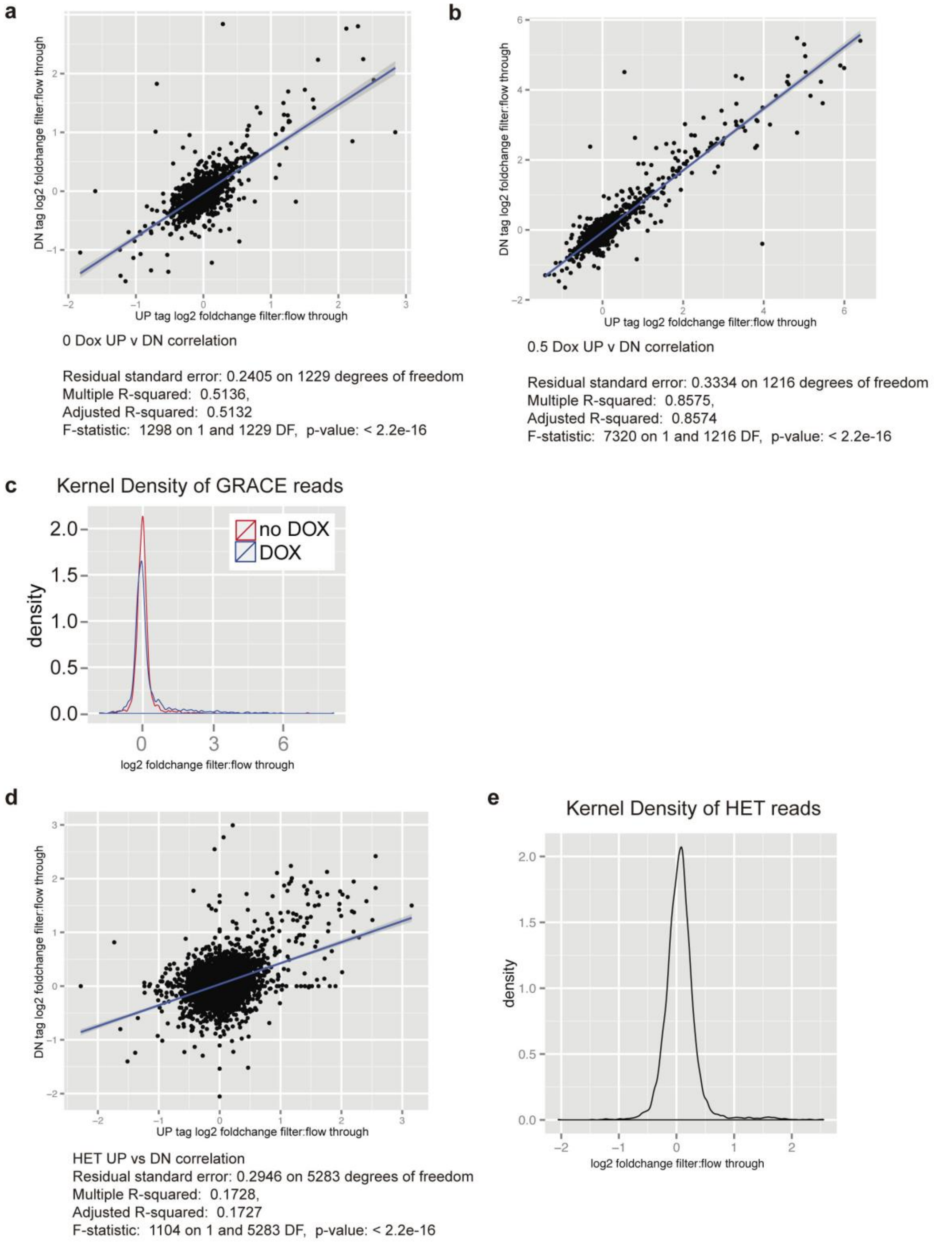

Supplementary Figure 4: Correlation between the $\log_2$ fold-change of normalized UPTAG and DNTAG barcode reads in the GRACE strains for the 0 μg mL^{-1} DOX **(A)** and 0.5 μg mL^{-1} DOX **(B)** treated pooled samples. **(C)** Kernal density graph of averaged

UPTAG and DNTAG log_2 reads in the GRACE strains for the 0 μg mL^{-1} DOX and 0.5 μg mL^{-1} DOX samples. **(D)** Correlation between the log_2 fold-change of normalized UPTAG and DNTAG barcode reads between filter retailed and flow-through HET strains. **(E)** Kernal density graph of averaged UPTAG and DNTAG log_2 reads in the HET strains.

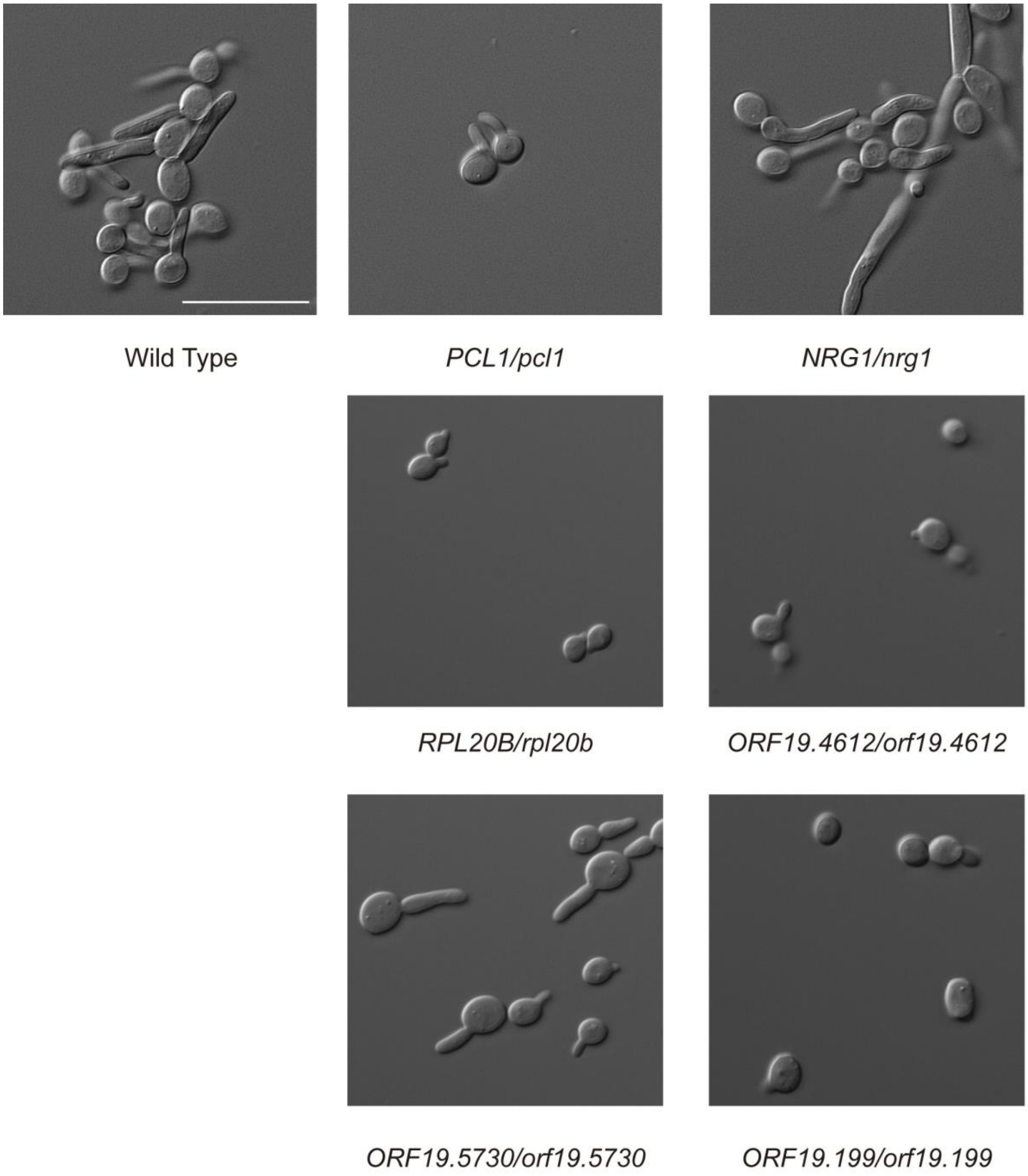

Supplementary Figure 5: Pooled analysis of the HET collection revealed mutants with altered morphogenesis. Phenotypes were verified after incubation for 30 minutes at 37°C in medium containing 1% v/v serum. All images taken at 40X magnification. Scale bar represents 20 μm.

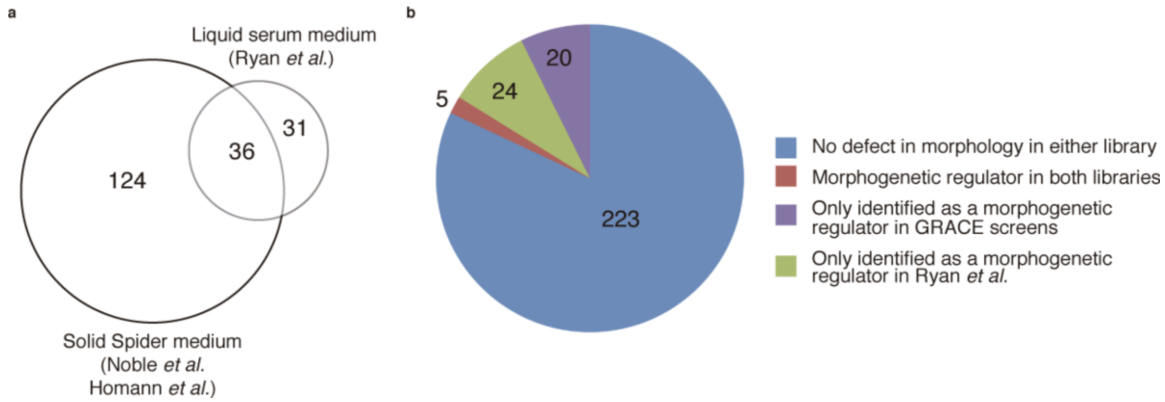

Supplementary Figure 6: Comparison of morphogenetic regulators identified in different studies. **(A)** Comparison of morphogenetic regulators identified in screens on Spider solid performed by Noble *et al.*[2] and Homann *et al.*[3] and those identified with the same libraries by Ryan *et al.*[6] in serum liquid conditions. (**B)** Comparison of morphogenetic regulators identified for mutants covered in the GRACE library and in the screens performed by Ryan *et al.*[4].

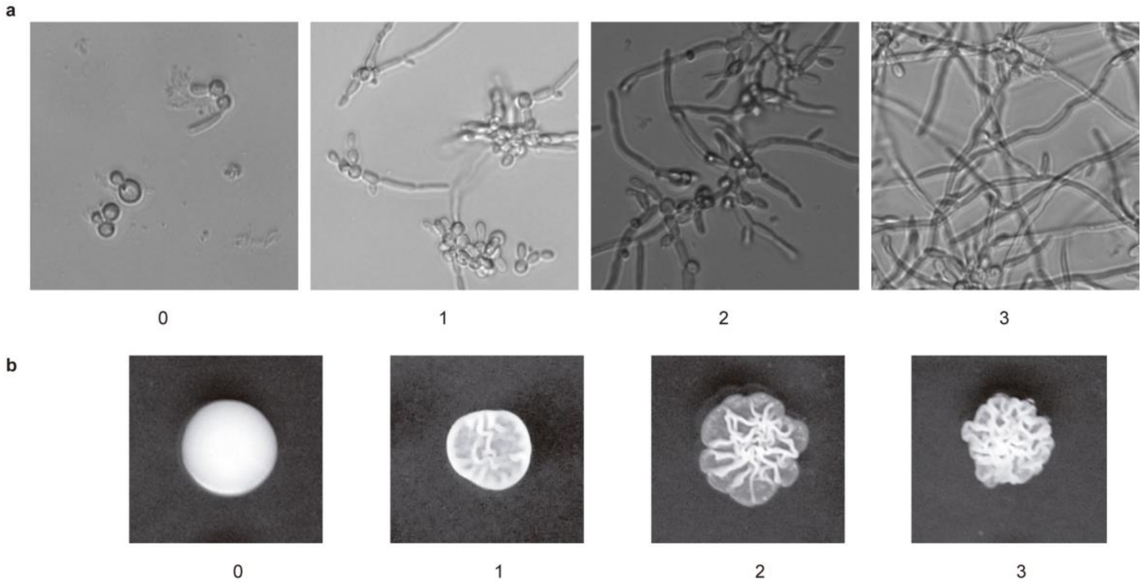

Supplementary Figure 7: (A) Microscopy images were scored from 0 to 3, based on the degree of filamentation. Representative images of each score are displayed here. (**B)** Solid plate filamentation was scored from 0 to 3, based on the colony morphology. Representative images of each score are displayed here.

Supplementary Table 1: Macrophage lysis and morphology scores

GRACE strain	No DOX		Plus DOX	
	Lysis Rate (%)	Morphology	Lysis Rate (%)	Morphology
TAF145	18.36	filament	0.61	pseudohyphae
BDF1	21.52	filament	0.69	yeast
orf19.1111	17.73	filament	0.74	short filaments
DBP5	19.14	filament	0.9	yeast
orf19.4882	17.65	filament	1.03	yeast
NPL6	14.6	filament	1.15	short filaments
VMA11	15.05	filament	2.17	yeast
VPS35	15.92	filament	2.59	filament
RFT1	18.1	filament	2.84	hyperfilament
SPC3	21.15	filament	3.03	yeast
ERG11	20.54	filament	3.39	pseudohyphae
CBF5	20.85	filament	3.48	yeast
PRE6	18.54	filament	3.61	pseudohyphae
RPO41	8.33	filament	4	yeast
ERG7	16.94	filament	4.36	yeast
SWP1	15.17	filament	4.38	yeast
ERG8	15.67	filament	4.41	yeast
STT3	17.73	filament	4.73	yeast
DQD1	23.51	filament	5.36	yeast
GUS1	23.32	filament	5.84	yeast
HSP60	22.77	filament	6.04	yeast
ERG6	25.27	filament	6.28	yeast
KRE9	18.1	filament	6.28	yeast
OST1	18.58	filament	6.38	yeast
SQT1	28.92	filament	6.75	short filaments
RAS1	21.09	filament	6.95	short filaments
ARC40	22.7	filament	7.65	pseudohyphae
PRP45	13.5	filament	8.14	yeast
WBP1	16.61	filament	8.2	yeast
NUS1	16.14	filament	8.28	yeast
GPI16	22.99	filament	8.33	yeast
IDH1	14.29	filament	8.41	filament
ARC15	23.14	filament	8.67	pseudohyphae
ALG14	15.26	filament	8.72	pseudohyphae
DEF1	21.58	filament	9.29	short filaments
ARC18	18.92	filament	9.68	pseudohyphae
CDC50	21.74	filament	9.7	yeast
ERG20	19.93	filament	9.7	yeast

SSS1	17.83	filament	9.95	yeast
HSP90	22.29	filament	10.32	short filaments
ENT2	23.4	filament	10.45	pseudohyphae
VPS53	22.64	filament	10.51	short filaments
MSL5	15.87	filament	10.55	yeast
OST2	20.15	filament	10.84	yeast
ALG7	18.9	filament	12.38	yeast
PAN1	18.69	filament	12.47	pseudohyphae
ALG11	12.9	filament	12.6	yeast
ECM31	21.64	filament	12.62	yeast
ARC35	22.16	filament	12.68	pseudohyphae
ARC19	12.88	filament	12.79	pseudohyphae
PMM1	19.87	filament	13.33	yeast
SAC3	17.02	filament	13.61	short filaments
orf19.6233	25.98	filament	15.98	yeast
CDC55	20.6	filament	16.67	pseudohyphae
TEP1	30.95	filament	21.67	filament
WT	24.63	filament	24.02	filament
ALG1	24.25	filament	24.51	yeast
BMT3	21.7	filament	25.19	filament
GPI10	23.65	filament	21.86	filament
GWT1	21.39	filament	20	short filaments
MCD4	19.33	filament	11.73	short filaments
MNN1	23.93	filament	24.6	short filaments
MNN24	21.94	filament	23.74	filament
MNT1	21.96	filament	23.51	filament
MNN2	26.29	filament	20.2	filament
OCH1	22.18	filament	23.53	short filaments
PMT1	16.52	filament	17.07	filament
VAN1	25.88	filament	24.67	filament

Supplementary Table 2: Macrophage lysis rates in response to pre-treatments and species

Strain	Pre-incubation	Treatment	Average Lysis Rate (%)	Standard Deviation	Comments
WT	YEPD	live	19.02	1.73	
WT	YEPD	heat killed	3.2	1.85	
WT	conditioned media	heat killed	3.11	0.49	
WT	30min phagocytosis	heat killed	6.34	1.67	
WT	60min phagocytosis	heat killed	17.51	4.84	
WT	60min phagocytosis	formalin killed	13.95	5.13	
WT	90min phagocytosis	heat killed	18.37	2.16	
WT	unphagocytized	heat killed	5.66	1.14	
WT	EndoH	heat killed	4.49	2.69	
ALG1	unphagocytized	heat killed	5.66	1.61	
ALG1	60min phagocytosis	heat killed	19.82	1.97	
ALG1	EndoH	heat killed	5.33	4.65	
orf19.6233	60min phagocytosis	heat killed	16.38	2.16	
orf19.6233	60min phagocytosis, fomalin killed	formalin killed	14.18	3.08	
orf19.6233	EndoH	heat killed	4.72	3.43	
OST1	unphagocytized	heat killed	1.38	1.96	
OST1	60min phagocytosis	heat killed	5.1	3.79	
ERG6	60min phagocytosis	heat killed	5.91	0.56	
S. cerevisiae	unphagocytized	heat killed	3	4.24	
S. cerevisiae	60min phagocytosis	heat killed	5.34	1.4	
C. neoformans WT	unphagocytized	heat killed	0	0	very few phagocytosis events
C. neoformans WT	60min phagocytosis	heat killed	2.56	3.63	very few phagocytosis events
C. neoformans cap59	YEPD	live	7.97	1.16	
C. neoformans cap59	unphagocytized	heat killed	1.6	0.8	
C. neoformans cap59	60min phagocytosis	heat killed	13.92	3.97	
C. neoformans cap59	EndoH	heat killed	1.56	2.21	

Supplementary Table 3: Oligonucleotides used in this study

Primer	Sequence (5' to 3')
oLC1131- *ERG11*- Forward	GATGTTTCTGCTGAAGATGC
oLC1132- *ERG11*- Reverse	ATAGTTGAGCAAATGAACGG
oLC2285- *ACT1*-Forward	GACCTTGAGATACCCAATTG
oLC2286- *ACT1*- Reverse	CAGCTTGAATGGAAACGTAG
oLC3546- *ALG1*-Forward	CTCAACTTCATTTACTCCTG
oLC3547- *ALG1*-Reverse	GTACTTTAGGATAGTCCTCTG
oLC3548- *PMM1*- Forward	CACCAATTGGTAGAAATGCT
oLC3549- *PMM1*- Reverse	GTTCATCTTCAACGTGTTGT
oLC3550- *ALG7*- Forward	TTTGTCGGAGTGTCATTGGC
oLC3551- *ALG7*- Reverse	CTTGGACAGGGCAAGATGTG
oLC3552- *ERG6*- Forward	TTATGCCATTGAAGCTACCG
oLC3553- *ERG6*- Reverse	GCTTGTTCAGCAACTTTACG
oLC3554- *ERG20*- Forward	TAGAAGGGGCCATTTATATC
oLC3555- *ERG20*- Reverse	CCAATTTGTTCTGGAGTACC
UPTAG universal amplification primer	AATGATACGGCGACCACCGAGATCTACACCGAGGTCGAGAATGATGTCCACGAGGTCTCT
UPTAG index amplification primer	CAAGCAGAAGACGGCATACGAGATNNNNNNGCCATTTGTCTGTCGACCTGCAGCGTACG
DNTAG universal amplification primer	AATGATACGGCGACCACCGAGATCTACACCACATGATATGTTGAGCGGTGTCGGTCTCGTAG
DNTAG index amplification primer	CAAGCAGAAGACGGCATACGAGATNNNNNNGAGTATCTGTATCTGGCC GAGCTCGAATTCATCGAT
UPTAG sequencing primer	CGAGGTCGAGAATGATGTCCACGAGGTCTCT
DNTAG sequencing primer	CACATGATATGTTGAGCGGTGTCGGTCTCGTAG
UPTAG index sequencing primer	CGTACGCTGCAGGTCGACAGACAAATGGC
DNTAG index sequencing primer	ATCGATGAATTCGAGCTCGGCCAGATACAGATACTC

Supplementary References:

1. Becker, J. M. *et al.* Pathway analysis of *Candida albicans* survival and virulence determinants in a murine infection model. *Proc Natl Acad Sci* **107,** 22044–22049 (2010).
2. Noble, S. M., French, S., Kohn, L. A., Chen, V. & Johnson, A. D. Systematic screens of a *Candida albicans* homozygous deletion library decouple morphogenetic switching and pathogenicity. *Nat Genet* **42,** 590–598 (2010).
3. Homann, O. R., Dea, J., Noble, S. M. & Johnson, A. D. A phenotypic profile of the *Candida albicans* regulatory network. *PLoS Genet* **5,** e1000783 (2009).
4. Ryan, O. *et al.* Global gene deletion analysis exploring yeast filamentous growth. *Science* **337,** 1353–1356 (2012).
